# CPSF6-Dependent Targeting of Speckle-Associated Domains Distinguishes Primate from Nonprimate Lentiviral Integration

**DOI:** 10.1128/mBio.02254-20

**Published:** 2020-09-29

**Authors:** Wen Li, Parmit K. Singh, Gregory A. Sowd, Gregory J. Bedwell, Sooin Jang, Vasudevan Achuthan, Amarachi V. Oleru, Doris Wong, Hind J. Fadel, KyeongEun Lee, Vineet N. KewalRamani, Eric M. Poeschla, Alon Herschhorn, Alan N. Engelman

**Affiliations:** a Department of Cancer Immunology and Virology, Dana-Farber Cancer Institute, Boston, Massachusetts, USA; b Department of Medicine, Harvard Medical School, Boston, Massachusetts, USA; c Department of Internal Medicine, Mayo Clinic, Rochester, Minnesota, USA; d Basic Research Laboratory, Center for Cancer Research, National Cancer Institute, Frederick, Maryland, USA; e Division of Infectious Diseases, University of Colorado Denver School of Medicine, Aurora, Colorado, USA; Columbia University/HHMI

**Keywords:** CPSF6, integration, LEDGF/p75, human immunodeficiency virus, lentiviruses

## Abstract

Lentiviral DNA integration favors transcriptionally active chromatin. We previously showed that the interaction of human immunodeficiency virus type 1 (HIV-1) capsid with cleavage and polyadenylation specificity factor 6 (CPSF6) localizes viral preintegration complexes (PICs) to nuclear speckles for integration into transcriptionally active speckle-associated domains (SPADs). In the absence of the capsid-CPSF6 interaction, PICs uncharacteristically accumulate at the nuclear periphery and target heterochromatic lamina-associated domains (LADs) for integration. The integrase-binding protein lens epithelium-derived growth factor (LEDGF)/p75 in contrast to CPSF6 predominantly functions to direct HIV-1 integration to interior regions of transcription units. Though CPSF6 and LEDGF/p75 can reportedly interact with the capsid and integrase proteins of both primate and nonprimate lentiviruses, the extents to which these different viruses target SPADs versus LADs, as well as their dependencies on CPSF6 and LEDGF/p75 for integration targeting, are largely unknown. Here, we mapped 5,489,157 primate and nonprimate lentiviral integration sites in HEK293T and Jurkat T cells as well as derivative cells that were knocked out or knocked down for host factor expression. Despite marked preferences of all lentiviruses to target genes for integration, nonprimate lentiviruses only marginally favored SPADs, with corresponding upticks in LAD-proximal integration. While LEDGF/p75 knockout disrupted the intragenic integration profiles of all lentiviruses similarly, CPSF6 depletion specifically counteracted SPAD integration targeting by primate lentiviruses. CPSF6 correspondingly failed to appreciably interact with nonprimate lentiviral capsids. We conclude that primate lentiviral capsid proteins evolved to interact with CPSF6 to optimize PIC localization for integration into transcriptionally active SPADs.

## Abstract (precis)

Integration is the defining step of the retroviral life cycle and underlies the inability to cure HIV/AIDS through the use of intensified antiviral therapy. The reservoir of latent, replication-competent proviruses that forms early during HIV infection reseeds viremia when patients discontinue medication. HIV cure research is accordingly focused on the factors that guide provirus formation and associated chromatin environments that regulate transcriptional reactivation, and studies of orthologous infectious agents such as nonprimate lentiviruses can inform basic principles of HIV biology. HIV-1 utilizes the integrase-binding protein LEDGF/p75 and the capsid interactor CPSF6 to target speckle-associated domains (SPADs) for integration. However, the extent to which these two host proteins regulate integration of other lentiviruses is largely unknown. Here, we mapped millions of retroviral integration sites in cell lines that were depleted for LEDGF/p75 and/or CPSF6. Our results reveal that primate lentiviruses uniquely target SPADs for integration in a CPSF6-dependent manner.

## INTRODUCTION

Integration links downstream steps of retrovirus replication to the transcriptional competence of the cell. Integration is mediated by viral integrase, which is a virion component. The preintegration complex (PIC), which is derived from the virion (1, 2), transports the integration machinery through the cell to sites of integration within the nucleus. Chromatin structure and functionality influence integration site selection, with viruses from different genera showing similar preferences for genes, transcriptional start sites (TSSs), local gene density, and transcriptional activity (see reference 3 for a recent review). Among studied retroviruses, integration targeting by lentiviruses and gammaretroviruses is most heavily influenced by host chromatin environment. HIV-1, a prototypical lentivirus, preferentially targets active genes (4) and speckle-associated domains (SPADs) (5), avoiding heterochromatin such as lamina-associated domains (LADs) (6–8). Moloney murine leukemia virus (MLV), the prototypical gammaretrovirus, also prefers genes for integration, though to a much lesser extent than HIV-1. In contrast to HIV-1, MLV integration favors TSSs and enhancer regions (9–11), which is primarily driven by the interaction of integrase with cellular bromodomain and extraterminal domain proteins (3).

The interaction of two viral proteins, integrase and capsid, with respective cellular proteins lens epithelium-derived growth factor (LEDGF)/p75 and cleavage and polyadenylation specificity factor (CPSF) 6 in large part determines HIV-1 integration targeting preferences. Although integration into genes is reduced by depleting either LEDGF/p75 (12–17) or CPSF6 (17), the two cofactors direct integration in different ways. CPSF6 regulates PIC positioning within the nucleus (reviewed in reference 18). In the absence of the capsid-CPSF6 interaction, PICs accumulate at the nuclear periphery (5, 8, 19–22). As a consequence, SPAD-proximal targeting is disfavored (5) and PICs uncharacteristically target LADs for integration (8). Loss of the LEDGF/p75-integrase interaction, in contrast, does not dramatically alter PIC positioning within the nucleus (8, 23, 24). Genic HIV-1 integration favors gene midbodies over 5′ and 3′ end regions (9, 16, 17). In the absence of LEDGF/p75, HIV-1’s genic integration pattern shifts toward gene 5′ regions (16, 17). LEDGF/p75 interacts with mRNA splicing factors (16) and can overcome the transcriptional block imposed by nucleosomes *in vitro* (25), potentially implicating mRNA splicing and/or transcriptional elongation in LEDGF/p75’s role in HIV-1 integration targeting.

While roles for LEDGF/p75 and CPSF6 in HIV-1 integration have been studied extensively, much less is known about how these factors influence the integration profiles of other retroviruses. LEDGF/p75 harbors two conserved domains, the N-terminal Pro-Trp-Trp-Pro (PWWP) domain important for chromatin binding and the integrase-binding domain (IBD) that is necessary and sufficient to bind HIV-1 integrase (26–29) (see Fig. S1A in the supplemental material). The integrase-LEDGF/p75 interaction is specific to the lentivirus genus of *Retroviridae* (30–32), and LEDGF/p75 depletion similarly reduced genic targeting by the nonprimate lentivirus equine infectious anemia virus (EIAV) and HIV-1 (14) while not significantly affecting MLV integration targeting (17). The role for LEDGF/p75 in the integration targeting of lentiviruses other than HIV-1 and EIAV has not been characterized.

10.1128/mBio.02254-20.1 FIG S1 LEDGF/p75 and CPSF6 domains and sequence conservation. (A) Shown below the map of LEDGF/p75 domains is length-matched percent identity plot across 6 mammalian species. Underneath is the amino acid sequence alignment of the IBD (human LEDGF/p75 residues 347 to 429) of the indicated LEDGF/p75 proteins, with amino acid hot spot residues Ile365, Asp366, and Phe406 for the interaction with HIV-1 integrase marked by asterisks. Index number is synonymous with amino acid position. CR, charged region. (B) CPSF6 domain organization highlights conserved RNA-recognition motif (RRM), proline-rich domain (PRD), and arginine/serine-rich-like domain (RSLD). Inset, amino acid sequence alignment of PRD region residues 276 to 290 (human CPSF6[551]), with hot spot residue Phe284 for the interaction with HIV-1 capsid marked by an asterisk. Download FIG S1, PDF file, 0.1 MB. Copyright © 2020 Li et al. 2020 Li et al. This content is distributed under the terms of the Creative Commons Attribution 4.0 International license.

CPSF6 is expressed as two isoforms, a minor one composed of 588 amino acids and the predominant protein composed of 551 residues (33, 34). CPSF6 was initially implicated in HIV-1 biology through the discovery of the C-terminal truncation CPSF6-358 mutant of CPSF6[588] that mislocalized to the cell cytoplasm and restricted PIC nuclear import (33). CPSF6-358 restricted infection by different primate lentiviruses including HIV-1, HIV-2, and simian immunodeficiency viruses derived from rhesus macaques (SIV_mac_ and SIV_mne_) but not infection by the felid lentivirus feline immunodeficiency virus (FIV) or MLV (33). An artificial construct composed of rhesus tripartite motif-containing (TRIM) 5 ring, B-box 2, and coiled-coil elements fused to CPSF6-358 similarly restricted infection by HIV-1 and SIV_mac_ without perturbing MLV, EIAV, FIV, or bovine immunodeficiency virus (BIV) infection (35). Hot spot CPSF6[551] residue Phe284 is important for the interaction with HIV-1 capsid (33, 36, 37) (Fig. S1B), but the relationship between binding and restriction is unclear because CPSF6_276–290_ peptide bound HIV-1, HIV-2, SIV_mac_, and FIV capsid N-terminal domain (NTD) proteins with similar affinities *in vitro* (37). As was observed for LEDGF/p75 depletion, CPSF6 knockout did not significantly affect MLV genic integration targeting (17). We are unaware of any reports that have documented a role for CPSF6 in the integration targeting of any retrovirus other than HIV-1.

Here, we investigate the relative contributions of LEDGF/p75 versus CPSF6 in integration targeting of primate and nonprimate lentiviruses using HEK293T and Jurkat T cell models. CPSF6 interactions with retroviral capsid proteins were assessed indirectly via CPSF6-358 restriction and biochemically using spectroscopy. Our results reveal that LEDGF/p75 plays similar important roles for genic integration targeting of all lentiviruses. However, only primate lentiviruses harbor a marked CPSF6-dependent preference to integrate into SPADs.

## RESULTS

### Evolutionary considerations.

This study focused on the following related research areas: first, to determine whether the marked integration preference of HIV-1 for SPADs (5) applied to other lentiviruses, and second, to assess the roles of LEDGF/p75 and CPSF6 in lentiviral integration targeting. Because the viruses under study were derived from an array of mammalian species, we analyzed the conservation of LEDGF/p75 and CPSF6 across representative animals including Homo sapiens, Macaca mulatta, Equus caballus, Felis catus, Bos taurus, and Mus musculus. The amino acid sequences of LEDGF/p75 orthologues were 88.6% identical (see Fig. S1A in the supplemental material) and 97.7% homologous considering conservative amino acid substitutions. Functionally critical PWWP domain and IBD regions were nearly identical, including IBD hot spot residues Ile365, Asp366, and Phe406 important for the interaction with HIV-1 integrase (38) (Fig. S1A). The only alteration among these regions, Ile in the IBD of *F. catus* LEDGF/p75 at the position analogous to human Val411, is unlikely to affect binding to FIV integrase because Val411 in IBD cocrystal structures with primate (39) and nonprimate (40) lentiviral proteins did not interact with these integrases. Human IBD protein carrying the V411A substitution, moreover, efficiently bound HIV-1 integrase *in vitro* (38). The CPSF6 orthologues were 99.3% identical and 99.6% homologous. Residues 276 to 290, which confer binding to HIV-1 capsid (36, 37), were identical across species (Fig. S1B). We conclude that human cells are a viable model to assess roles of LEDGF/p75 and CPSF6 in integration targeting of mammalian retroviruses.

### Viral infection of HEK293T cells.

**FIG 1:**
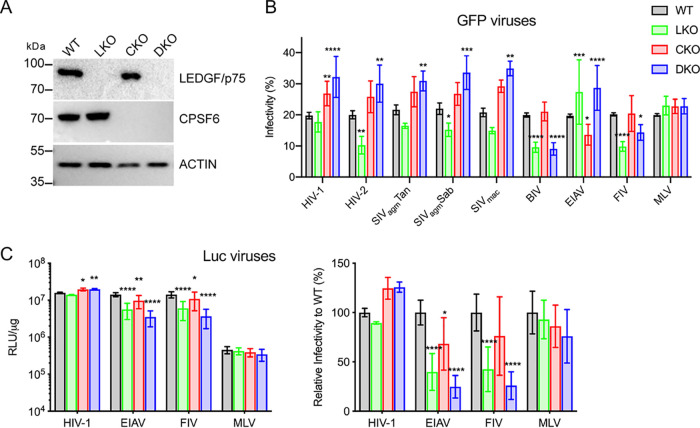
Virus infection of HEK293T cells. (A) Western immunoblots using anti-LEDGF/p75 and anti-CPSF6 antibodies; β-actin was blotted as a loading control. Numbers to the left are mass marker positions in kDa. (B) Infectivities of indicated GFP reporter viruses with WT, LKO, CKO, and DKO cells. (C) Left, infectivities of Luc reporter viruses in relative light units per μg total protein (RLU/μg). To highlight differences among cell types, the data were replotted by normalizing each virus with WT cells to 100% (rightward graph). Results (average ± standard deviation [SD]) compile data from at least four independent experiments, with each experiment conducted in duplicate. Significant differences in comparison to results observed with WT cells are indicated: *, *P* < 0.05; **, *P* < 0.01; ***, *P* < 0.001; ****, *P* < 0.0001.

Single-round retroviral vectors capable of expressing green fluorescent protein (GFP) or firefly luciferase (Luc) reporters were pseudotyped by cotransfection with vesicular stomatitis virus glycoprotein G (VSV-G). Infection profiles of GFP-expressing lentiviruses versus MLV were initially assessed on wild-type (WT) HEK293T cells as well as isogenic derivatives that were knocked out for LEDGF/p75 (LKO for LEDGF knockout), CPSF6 (CKO), or both factors (DKO for double knockout) (17, 41) (Fig. 1A). Preliminary experiments determined levels of viral inocula that conferred ∼20% GFP positivity to WT cells at 48 h postinfection, equating to approximate multiplicities of infection (MOIs) of 0.2 (Fig. 1B, gray bars). The abilities of HIV/SIV viruses to infect LKO cells were impaired ∼10% to 50% compared to the levels at which they infected WT cells (Fig. 1B). While BIV-GFP and FIV-GFP infected LKO cells about 2-fold less efficiently than WT cells, EIAV-GFP infectivity was increased by ∼40%. Primate lentiviruses infected CKO cells more efficiently than WT cells (Fig. 1B), as demonstrated previously for HIV-1 (17, 33, 42). BIV and FIV infected WT and CKO cells at indistinguishable levels, while EIAV-GFP infection was marginally reduced. Similar to their titers on CKO cells, primate lentiviruses infected DKO cells more efficiently than WT cells. While the titer of EIAV was also enhanced on DKO compared to WT cells, BIV and FIV infected DKO cells less efficiently than WT cells. As expected (13, 17, 41), MLV-GFP similarly infected all cell types (Fig. 1B).

LEDGF/p75 was required for optimal infection of mouse cells by a separate EIAV-GFP construct (14). To increase the sensitivity of virus detection, a Luc derivative of EIAV-GFP was tested alongside other Luc-encoding viruses including HIV-Luc, FIV-Luc, and MLV-Luc. The infectivities of HIV-Luc with the different HEK293T cell types mirrored those observed for HIV-GFP (Fig. 1B and C). FIV-Luc infection of LKO and DKO cells was reduced by ∼60% and 75%, respectively, compared to WT cells. Because EIAV-Luc infection was similarly reduced on these respective cell types, we inferred that EIAV’s behavior with LKO and DKO cells could in part be reporter gene dependent. Matched EIAV-GFP versus EIAV-Luc doses across a 128-fold MOI range confirmed that the GFP reporter in large part masked EIAV’s dependence on LEDGF/p75 for efficient HEK293T cell infection (Fig. S2). EIAV infectivities by comparison were reduced under most conditions of CKO cell infection (Fig. 1B and C and Fig. S2).

10.1128/mBio.02254-20.2 FIG S2 Dose escalation of EIAV infections of HEK293T cell types. Titration of matched reverse transcriptase (RT) activity units of EIAV-GFP (top panels) versus EIAV-Luc with indicated cell types. Raw data (left panels) were replotted to the right, percent normalized for virus activity on WT cells at each virus dose. Results (average ± SD) compile data from two independent experiments, with each experiment conducted in duplicate. Significant differences in comparison to results observed with WT cells are indicated: **, *P* < 0.01; ***, *P* < 0.001; ****, *P* < 0.0001. Download FIG S2, PDF file, 0.5 MB. Copyright © 2020 Li et al. 2020 Li et al. This content is distributed under the terms of the Creative Commons Attribution 4.0 International license.

### Jurkat T cell models and virus infection.

**FIG 2:**
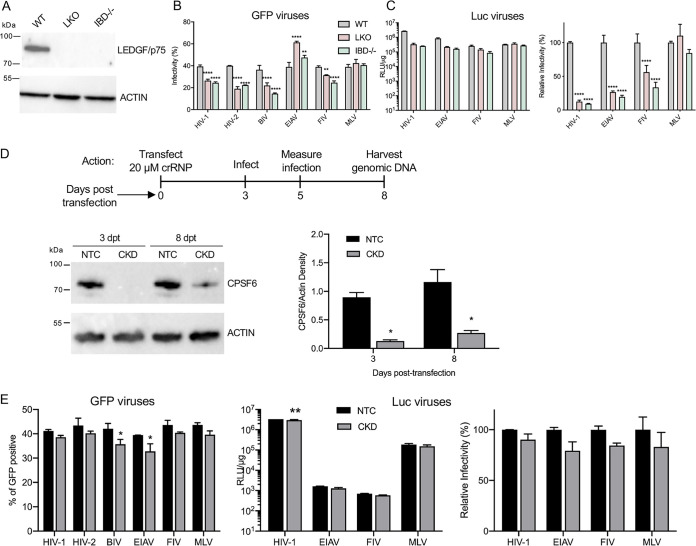
Virus infection of Jurkat T cells. (A) Western immunoblot using anti-LEDGF/p75 antibody; β-actin was blotted as a loading control. Numbers to the left are mass marker positions. (B) Infectivities of indicated GFP reporter viruses with WT, LKO, and IBD^−/−^ cells. (C) Luc reporter virus infectivities. To highlight differences among cell types, the data were replotted to the right as percent normalized to the level of virus infection with WT cells. (D) Experimental timeline of CPSF6 depletion by crRNP transfection. Lower left is a representative immunoblot of cell lysates made at 3 and 8 days posttransfection (dpt). The graph to the right shows average CPSF6 band intensities normalized to β-actin for two independent blotting experiments (± standard error of the mean). (E) Infectivities of indicated GFP (leftward graph) and Luc reporter viruses with Jurkat T cells that had been transfected with NTC versus *CPSF6*-targeting crRNPs. To highlight differences among cell types, Luc viral data were replotted as percent normalized to the level of virus infection with WT cells (right). RLU, relative light units. Results (average ± SD) compile data from at least two independent infection experiments, each conducted with technical duplicates. Significant differences in comparison to results observed with WT or NTC-transfected cells are indicated: *, *P* < 0.05; **, *P* < 0.01; ****, *P* < 0.0001.

Two previously described Jurkat T cell LKO derivatives engineered using transcription activator-like effector nuclease (TALEN) technology were analyzed initially. Whereas the majority of the *PSIP1* gene, which encodes LEDGF/p75, was deleted from one cell line, the other harbored deletions of exons 12 to 14, which encode the protein’s IBD region (41). Here, we refer to these cells as LKO and IBD^−/−^ (Fig. 2A).

Preliminary experiments determined levels of GFP reporter viruses required to transduce ∼40% of WT Jurkat T cells, corresponding to approximate MOIs of 0.5 (Fig. 2B, gray bars). Whereas HIV-1, HIV-2, BIV, and FIV infected LKO and IBD^−/−^ cells ∼20% to 60% less efficiently than WT cells, EIAV-GFP, as was observed with HEK293T LKO cells, infected these cells ∼20% to 50% more efficiently than WT cells (Fig. 2B). Also similar to the work with HEK293T cells, Luc-encoding lentiviruses revealed comparably greater infectivity defects than did their GFP counterparts (compare Fig. 2C and B). MLV infected LKO and IBD^−/−^ cells at levels comparable to WT Jurkat T cells (Fig. 2B and C).

Despite extensive effort, we were unable to create Jurkat T cell clones knocked out for CPSF6. We accordingly devised a transient knockdown strategy to deplete CPSF6 expression levels. Cells transfected with *CPSF6*-targeting or nontargeting control (NTC) CRISPR RNA (crRNA)-Cas9 ribonucleoprotein complexes (crRNPs) were infected at 3 days posttransfection (dpt). As before, levels of virus infection were assessed 2 days thereafter. These experiments were terminated at 8 dpt, at which time genomic DNA was prepared for integration site analyses (see below). Semiquantitative immunoblotting revealed that the expression level of CPSF6 was reduced to ∼14% and 25% of the corresponding NTC cell level at 3 dpt and 8 dpt, respectively (Fig. 2D).

Most retroviruses infected CPSF6 knockdown (CKD) and NTC cells similarly. Of note, we did not observe increased levels of HIV infection that can be observed when CPSF6 is depleted from other cell types (17, 33, 42) (Fig. 1B and C). In comparison to cells transfected with NTC crRNPs, CKD marginally impaired BIV-GFP, EIAV-GFP, and HIV-Luc infection (Fig. 2E).

### Integration sites in HEK293T cells.

Cellular DNAs isolated 5 days postinfection were amplified using ligation-mediated (LM)-PCR for sequencing on the Illumina platform. Resulting sequences were parsed bioinformatically to yield unique integration sites, which were mapped with respect to RefSeq genes, SPADs, local gene density (±0.5 Mb of integration site), and proximity (±2.5 kb) to TSSs and LADs. For each virus, a random integration control (RIC) was generated *in silico* based on the genomic DNA fragmentation technique. As one example, DNA isolated from HIV-1-infected cells was digested with MseI and BglII. The corresponding RIC was generated by fragmenting human draft genome hg19 at MseI and BglII sites and then mapping these in parallel with Illumina sequencing reads. Due to similar infection phenotypes of primate lentiviral GFP constructs (Fig. 1B), integration sites were mapped for HIV-1 and HIV-2 as representative primate lentiviruses. These profiles were compared to nonprimate lentiviruses BIV, EIAV, and FIV, as well as MLV, which served as control. Across HEK293T cell types, 747,130 unique integration sites were mapped (Table 1). In total, 6,506,678 integration sites (5,489,157 lentiviral and 1,017,471 MLV) were mapped in this study.

**TABLE 1:** Integration distributions in HEK293T cells with respect to defined genomic annotations^*a*^

Virus^*b*^	Cell type^*c*^	Unique sites	In RefSeq genes (%)	±2.5-kb TSS (%)	Gene density ±500 kb	±2.5-kb LAD (%)	In SPADs (%)
HIV-1	WT	20,461	82.9	3.9	21.3	18.7	31.5
	LKO	10,702	62.2	9.1	14.9	28.9	17.2
	CKO	33,056	60.8	1.8	6.5	59.8	0.9
	DKO	22,779	44.6	4.7	7.2	59.0	2.7
	RIC	112,183	45.1	3.3	7.9	51.2	2.8

HIV-2	WT	45,619	78.2	4.5	20.7	17.6	29.6
	LKO	30,497	58.3	13.5	15.6	27.1	19.3
	CKO	72,472	62.8	2.1	7.3	53.7	1.5
	DKO	13,931	43.3	6.6	7.5	56.6	3.0
	RIC	28,188	45.3	3.5	7.9	51.0	2.8

BIV	WT	131,650	70.4	3.0	10.8	38.5	6.1
	LKO	32,676	58.4	6.3	10.2	42.5	6.2
	CKO	86,841	67.4	2.9	10.7	38.3	6.0
	DKO	28,616	47.4	8.7	9.1	48.8	5.4
	RIC	112,396	45.3	3.4	7.9	51.3	2.8

EIAV	WT	7,673	78.5	3.3	14.8	28.3	14.1
	LKO	1,082	55.2	11.6	13.2	35.3	12.4
	CKO	5,593	74.5	3.9	14.4	28.0	13.5
	DKO	1,272	54.2	9.1	12.2	33.7	11.0
	RIC	27,903	45.2	3.5	8.0	50.6	3.2

FIV	WT	56,336	66.0	2.8	9.3	42.3	3.3
	LKO	5,144	54.4	7.0	11.7	34.2	8.7
	CKO	52,991	64.2	2.5	9.0	43.6	3.1
	DKO	15,452	47.7	7.0	11.1	36.8	9.1
	RIC	28,132	45.1	3.4	7.9	51.1	2.9

MLV	WT	14,526	60.8	54.0	17.5	22.1	24.2
	LKO	16,879	58.6	62.3	17.1	22.7	22.7
	CKO	16,181	60.8	63.1	18.0	21.3	26.1
	DKO	24,701	53.7	52.4	16.9	24.3	23.4
	RIC	112,183	45.1	3.2	7.9	51.2	2.8

a Results from two independent sets of infections; see Table S1 for accompanying statistical analyses.

b GFP viral constructs.

c RIC, random integration control.

10.1128/mBio.02254-20.6 TABLE S1 Statistical analyses of integration site distributions in the human genome. Download Table S1, XLSX file, 0.04 MB. Copyright © 2020 Li et al. 2020 Li et al. This content is distributed under the terms of the Creative Commons Attribution 4.0 International license.

**FIG 3:**
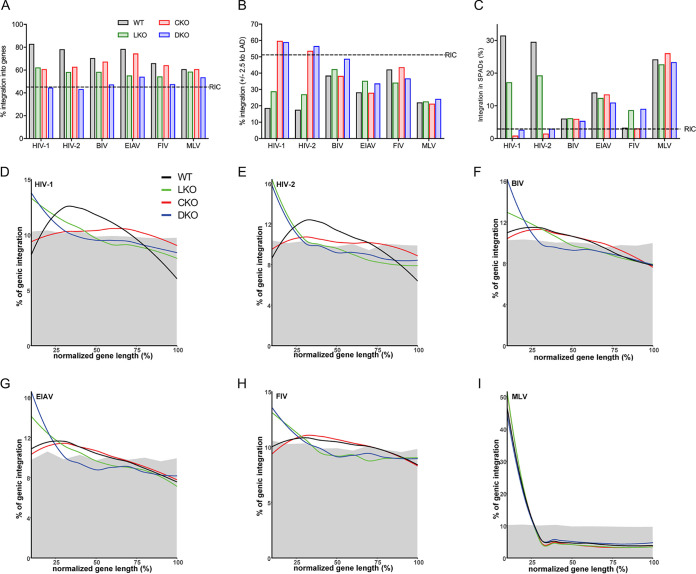
Retroviral integration site distributions in HEK293T cells. (A) Integration frequencies in WT or indicated KO cells with respect to RefSeq genes. RIC represents the average value (45.2%) among studied viruses (Table 1). (B) Integration frequencies near (±2.5 kb) LADs. RIC is the average 51.1% value across data sets. (C) SPAD integration frequencies. The 2.9% RIC value is the average across data sets. See Table S1 for corresponding statistical analyses. (D to I) Integration patterns of indicated viruses along all targeted genes, percent normalized for length. Gray shades highlight approximate 10% RIC values.

As expected, the genic integration frequency of HIV-1 in WT cells, 82.9%, was highly enriched compared to random (Fig. 3A and Table 1; see Table S1 for corresponding *P* values). Consistent with prior work (17), genic HIV-1 integration was reduced significantly, by ∼20.7% and 22.1%, in LKO and CKO cells, respectively. The extent of gene targeting in DKO cells, 44.6%, was, moreover, indistinguishable from random (*P* = 0.39). Similar trends applied to HIV-2, where in the absence of LEDGF/p75 or CPSF6 genic targeting was reduced by ∼19.9% and 15.4%, respectfully; in this case, gene targeting in DKO cells was marginally disfavored (*P* = 0.02). From these analyses, we conclude that LEDGF/p75 and CPSF6 each play a major role to direct HIV-1 and HIV-2 integration into genes and that genic targeting is counteracted by the combined depletion of the two cofactors. As reported previously (17), gene targeting by MLV was largely unaffected by LEDGF/p75 or CPSF6 depletion (Fig. 3A).

LEDGF/p75 played a similarly important role in gene targeting by BIV, EIAV, and FIV (Fig. 3A and Table 1). In contrast, loss of CPSF6 reduced BIV, EIAV, and FIV integration into genes by only ∼1.8% to 4.0%. While the level of genic targeting by EIAV was indistinguishable in LKO and DKO cells (*P* = 0.81), BIV and FIV genic integration levels in DKO cells were reduced significantly from their respective levels in LKO cells (Fig. 3A).

In contrast to MLV, HIV-1 displays minimal preference for promoter-proximal integration (Table 1 and Table S1) (9, 13). However, integration into promoters increased (*P* = 1.4 × 10^−65^) and decreased (*P* = 2.1 × 10^−45^) significantly in the absence of LEDGF/p75 and CPSF6, respectively (17), trends that likewise applied to HIV-2 (Table 1 and Table S1). Similarly, in the absence of LEDGF/p75, TSS-proximal integration increased for BIV (*P* = 1.9 × 10^−137^), EIAV (*P* = 2.6 × 10^−24^), and FIV (*P* = 4.3 × 10^−46^). Yet, in stark contrast to HIV-1 and HIV-2, integration frequencies near promoters remained largely unchanged for BIV and EIAV (*P* = 0.13 for both) and FIV (*P* = 0.04) in the absence of CPSF6 (Table S1).

HIV-1 integration disfavors LADs (7, 8) and favors SPADs (5). Although LEDGF/p75 played a significant role in these targeting preferences, HIV-1 in LKO cells continued to disfavor LADs and favor SPADs (5, 8) (Fig. 3B and C). In the absence of CPSF6, these phenotypes reversed such that HIV-1 integration favored LADs and disfavored SPADs (5, 8). HIV-2 integration in WT cells also significantly favored SPADs and disfavored LADs. In CKO cells, HIV-2 revealed overall similar behavior as HIV-1, uncharacteristically favoring LADs (*P* = 1.1 × 10^−5^) and disfavoring SPADs (*P* = 5.0 × 10^−39^) (Fig. 3B and C). Because DKO cell phenotypes were more similar to CKO than to LKO cells, we conclude CPSF6’s role in HIV-1/2 integration is to primarily counteract LAD targeting and promote SPAD targeting.

Although BIV, EIAV, and FIV integration also disfavored LADs in WT cells, the magnitudes of these effects, which ranged from ∼8.8% for FIV to 22.3% for EIAV versus respective RICs, were noticeably less than the ∼33% differences observed for HIV-1 and HIV-2 (Fig. 3B). While loss of LEDGF/p75 incurred overall similar changes in LAD-proximal targeting across lentiviruses, the primate and nonprimate viruses responded noticeably differently to loss of CPSF6. In stark contrast to HIV-1 and HIV-2, LAD-proximal integration targeting by BIV (*P* = 0.55), EIAV (*P* = 0.78), and FIV (*P* = 0.006) was largely similar in WT and CKO cells. LAD-proximal integration targeting frequencies of MLV were similar in WT, LKO (*P* = 0.29), and CKO (*P* = 0.18) cells.

Largely similar behaviors were observed among retroviral SPAD-targeting phenotypes. Although BIV, EIAV, and FIV integration favored SPADs, the magnitudes of these effects were noticeably less than those observed for HIV-1 and HIV-2. Moreover, CPSF6 played little to no role in SPAD-tropic integration targeting of BIV (*P* = 0.37), EIAV (*P* = 0.36), and FIV (*P* = 0.02) (Fig. 3C).

Mapping integrations across genes can highlight genus-specific differences (9) and inform roles of cofactors in targeting preferences (17). For example, the preference for HIV-1 to target gene midregions (9) shifted to 5′ end regions in the absence of LEDGF/p75 (16, 17), while CPSF6 knockout reduced integration to a fairly consistent level along gene lengths (17). As the integration distribution in DKO cells mirrored the LKO cell distribution, we previously concluded that a main function of LEDGF/p75 is to guide HIV-1 integration to gene midregions (17). Analysis of other viral integration patterns revealed this as a conserved lentiviral phenotype (Fig. 3D to I). The HIV-2 length normalized CKO curve appeared similar to the associated HIV-1 trace (compare Fig. 3E and D). The EIAV, FIV, and BIV curves in CKO cells by contrast appeared similar to their respective curves in WT cells, which is consistent with the comparatively minor role for CPSF6 in genic integration targeting by these viruses (Fig. 3A). As expected (9), the length normalized MLV curve in WT cells spiked sharply in the 5′ end region, with only minor deviations noticeable across cell types (Fig. 3I).

### Integration sites in Jurkat T cells.

Preliminary analysis of HIV-1 integration in WT, LKO, and IBD^−/−^ Jurkat T cells revealed what appeared to be a more potent retargeting phenotype in IBD^−/−^ than LKO cells. Integration frequencies in genes and SPADs, as well as the average number of genes/Mb surrounding integration sites, were each reduced more significantly in IBD^−/−^ cells than in LKO cells (Table 2 and Table S1). Conversely, upticks in TSS- and LAD-proximal integration typical for the loss of LEDGF/p75 were more pronounced in IBD^−/−^ than in LKO cells. Based on these findings, additional LKO cell lines were generated and characterized. Similarly to the original IBD^−/−^ strategy, exon 12 of *PSIP1* was targeted, though in this case via CRISPR-Cas9 (Fig. S3A). Following transduction with LentiCRISPRv2 constructs, cells were cloned by limiting dilution and single-cell clones were typed via DNA sequencing (Fig. S3B) and immunoblotting (Fig. S3C). Clones 1-F10 and 2-C10, each of whose exon 12 alterations resulted in LEDGF/p75 truncation amid the IBD (Fig. S3B), were selected for detailed analysis. Lentiviral GFP and Luc viruses infected 1-F10 and 2-C10 cells at levels that were reduced significantly from the levels at which they infected a control cell line that was cloned following transduction with a nontargeting (NT) lentiviral vector. MLV in contrast similarly infected NT, 1-F10, and 2-C10 cells, confirming the LKO cell phenotype (Fig. S3D). Extents of genic HIV-1 integration targeting in 1-F10 and 2-C10 cells, 52.0% and 53.0%, respectively, were similar to the 53.1% value observed in IBD^−/−^ cells (compare Fig. S3E and Table 2). Based on this, our retroviral integration site analyses focused on IBD^−/−^ Jurkat T cells.

**TABLE 2:** Integration distributions in WT and LKO Jurkat T cells^*a*^

Virus^*b*^	Cell type^*c*^	Unique sites	In RefSeq genes (%)	±2.5-kb TSS (%)	Gene density ±500 kb	±2.5-kb LAD (%)	In SPADs (%)
HIV-1	WT	234,077	81.8	4.7	21.1	17.0	30.8
	LKO	40,089	59.6	7.7	16.2	27.5	21.2
	IBD^−/−^	62,438	53.1	8.3	14.4	31.3	17.4
	RIC	112,183	45.1	3.3	7.9	51.2	2.8

HIV-2	WT	484,800	81.6	4.7	19.0	17.2	24.6
	LKO	53,999	69.3	6.3	17.2	22.5	21.8
	IBD^−/−^	65,715	52.6	9.7	14.7	29.1	18.0
	RIC	28,188	45.3	3.5	7.9	51.0	2.8

BIV	WT	112,596	72.0	3.2	10.3	35.6	4.1
	LKO	29,138	55.6	4.4	9.2	41.3	4.1
	IBD^−/−^	31,824	45.4	4.7	8.7	45.5	4.8
	RIC	112,396	45.3	3.4	7.9	51.3	2.8

EIAV	WT	25,362	68.7	3.4	10.9	32.6	5.4
	LKO	12,719	52.2	5.1	10.8	37.3	6.8
	IBD^−/−^	22,072	46.7	5.4	10.3	39.4	6.9
	RIC	27,903	45.2	3.5	8	50.6	3.2

FIV	WT	182,307	79.3	2.5	9.6	36.7	2.3
	LKO	50,931	71.3	3.5	9.8	37.5	3.5
	IBD^−/−^	31,630	53.7	5.4	10.0	40.2	6.3
	RIC	28,132	45.1	3.4	7.9	51.1	2.9

MLV	WT	264,353	53.9	22.5	13.1	26.5	12.4
	LKO	123,736	54.1	29.0	14.6	24.6	16.7
	IBD^−/−^	269,519	51.9	20.6	14.1	25.6	15.7
	RIC	112,183	45.1	3.3	7.9	51.2	2.8

a Results from one set of duplicate infections; see Table S1 for accompanying statistical analyses.

b GFP viral constructs.

c IBD, integrase-binding domain; RIC, random integration control.

10.1128/mBio.02254-20.3 FIG S3 CRISPR-Cas9-mediated targeting of *PSIP1*. (A) *PSIPI* gene locus on human chromosome 9 highlights the region of sgRNA targeting. (B) LEDGF/p75 protein alterations (red font) inferred from sequencing cloned exon 12 amplicons. Hot spot interaction residues I365, D366, and F406 are underlined. Asterisks, stop codons; h, alpha helices. (C) Immunoblot of indicated Jurkat cell lysates; β-actin was blotted as a loading control. Numbers to the left are mass marker positions in kDa. (D) Infectivities of GFP and Luc viral constructs with the indicated cell line (average ± SD for *n* = 2 independent experiments, with each experiment conducted in duplicate). Luc viral activity levels were normalized to their levels with NT cells, which were set to 100%. ****, *P* < 0.0001. (E) Mapped HIV_NLX.Luc.R-U3-Tag_-Luc integration sites in indicated cell lines; the EIAV U3-tagged virus affords integration site determinations in cells that harbor preexisting integrated LentiCRISPRv2 (66). See Table S1 for associated statistical analyses. Download FIG S3, PDF file, 1.0 MB. Copyright © 2020 Li et al. 2020 Li et al. This content is distributed under the terms of the Creative Commons Attribution 4.0 International license.

**FIG 4:**
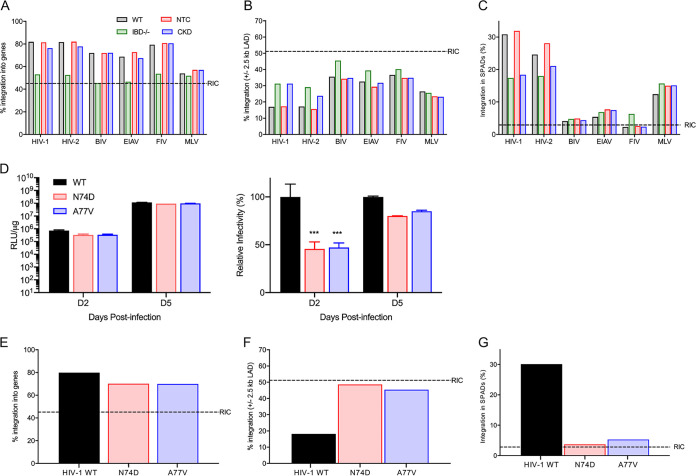
Retroviral integration site distributions in Jurkat T cells. (A) Integration frequencies for the indicated viruses with respect to RefSeq genes. IBD^−/−^ data from Table 2 (green) are compared to WT control (gray); CKD data in blue are compared with NTC (red) from Table 3. (B) Integration frequencies near LADs. (C) SPAD integration profiles among studied viruses. (D) Left, infectivities of N74D and A77V Luc reporter viruses in RLU/μg. To highlight differences between conditions, the data were replotted with the activity of WT HIV-Luc at each time point set to 100% (rightward graph). Results (average ± SD) compile data from two independent experiments, with each experiment conducted in duplicate. Significant differences in comparison to WT HIV-Luc are indicated (***, *P* < 0.001). (E to G) Capsid mutant viral integration frequencies with respect to RefSeq genes (E), LADs (F), and SPADs (G) (Table 4; see Table S1 for statistical analyses). The legend to Fig. 3 defines RIC values for panels A to C and panels E to G.

Genic integration targeting by lentiviruses was impressively reduced, by 22.0% to 29.0%, in IBD^−/−^ compared to WT Jurkat T cells (Table 2 and Fig. 4A). For BIV, genic targeting in IBD^−/−^ cells was indistinguishable from random (*P* = 0.76). HIV-1, HIV-2, and EIAV genic targeting was reduced significantly, by 5.2%, 4.2%, and 5.4%, respectively, in CKD cells (Table 3). Conversely, gene targeting by BIV (*P* = 0.62), FIV (*P* = 0.41), and MLV (*P* = 0.72) was unaffected in CKD cells.

**TABLE 3:** Integration distributions in WT and knockdown Jurkat T cells^*a*^

Virus^*b*^	Cell type^*c*^	Unique sites	In RefSeq genes (%)	±2.5-kb TSS (%)	Gene density ±500 kb	±2.5-kb LAD (%)	In SPADs (%)
HIV-1	NTC	159,125	81.5	5.2	21.2	17.3	31.9
	CKD	118,723	76.3	3.9	15.3	31.3	18.4
	RIC	112,183	45.1	3.3	7.9	51.2	2.8

HIV-2	NTC	393,694	82.0	5.4	20.2	15.6	28.1
	CKD	266,104	77.8	4.6	17.0	23.8	21.1
	RIC	28,188	45.3	3.5	7.9	51.0	2.8

BIV	NTC	127,706	72.0	3.7	10.7	34.3	4.9
	CKD	130,411	72.2	3.5	10.5	34.8	4.4
	RIC	112,396	45.3	3.4	7.9	51.3	2.8

EIAV	NTC	18,464	72.8	3.8	12.2	29.4	7.7
	CKD	34,387	67.4	3.8	11.8	31.8	7.5
	RIC	27,903	45.2	3.5	8.0	50.6	3.2

FIV	NTC	313,481	80.8	2.8	10.0	34.8	2.6
	CKD	308,420	80.6	2.7	9.9	34.9	2.4
	RIC	28,132	45.1	3.4	7.9	51.1	2.9

MLV	NTC	160,057	57.1	25.6	14.2	23.5	15.0
	CKD	127,519	57.0	25.1	14.3	23.2	15.1
	RIC	112,183	45.1	3.3	7.9	51.2	2.8

a Results from one set of duplicate infections; see Table S1 for accompanying statistical analyses.

b GFP viral constructs.

c NTC, nontargeting control; CKD, CPSF6 knockdown; RIC, random integration control.

Magnitude changes in lentiviral LAD- and SPAD-tropic integration targeting were similar in Jurkat T and HEK293T LKO cells compared to WT cells (compare Fig. 4B and C and Fig. 3B and C). LEDGF/p75 loss also shifted sites of genic integration toward gene 5′ end regions in Jurkat T cells (Fig. S4A to F). Despite the comparatively mild effect of CKD versus CKO on genic integration targeting by primate lentiviruses, CKD nonetheless significantly reduced HIV-1 and HIV-2 integration into SPADs (*P* < 10^−300^ for both). Strikingly, the 5.4% downshift in genic integration for EIAV in CKD cells was not accompanied by a corresponding change in SPAD targeting (*P* = 0.64).

10.1128/mBio.02254-20.4 FIG S4 Normalized genic integration patterns in Jurkat T cells. (A to F) Integration patterns of indicated viruses along all targeted genes, percent normalized for length. IBD^−/−^ is compared to WT while CKD is compared to NTC. (G) Normalized genic integration pattern of N74D and A77V capsid mutant viruses versus WT HIV-Luc. Gray shades highlight approximate 10% RIC values. Download FIG S4, PDF file, 0.7 MB. Copyright © 2020 Li et al. 2020 Li et al. This content is distributed under the terms of the Creative Commons Attribution 4.0 International license.

The 5.2% reduction in genic HIV-1 integration targeting in Jurkat CKD cells was noticeably less than the 22.1% reduction in CKO cells (compare Fig. 4A and Fig. 3A). While the reason for this difference is unclear, it seemed possible that the residual level of CPSF6 protein that persisted in CKD cells (Fig. 2D) could be a contributing factor. We accordingly next mapped integration sites of N74D and A77V capsid mutant viruses in Jurkat T cells. N74D and A77V capsid nanotubes bound CPSF6 ∼5% and 30%, respectively, as efficiently as WT capsid complexes *in vitro* (43).

The infection levels of N74D and A77V viruses in Jurkat T cells were reduced ∼50% versus WT HIV-Luc after 2 days. Three days later, when DNA was harvested for sequencing, the infection levels of all three viruses were statistically indistinguishable (Fig. 4D). Genic integration targeting by the mutant viruses was reduced ∼10% compared to the WT virus (Fig. 4E and Table 4) with gene length-normalized profiles more closely resembling the WT virus in CKO than in CKD cells (compare Fig. 3D with Fig. S4A and G). The capsid changes nearly ablated SPAD targeting, with corresponding upticks in LAD-proximal integration (Fig. 4F and G and Table 4).

**TABLE 4:** Integration distributions of capsid mutant viruses in Jurkat T cells^*a*^

HIV-1^*b*^	Unique sites	In RefSeq genes (%)	±2.5-kb TSS (%)	Gene density ±500 kb	±2.5-kb LAD (%)	In SPADs (%)
WT	562,503	79.7	5.0	20.6	18.2	30.1
N74D	473,247	70.0	2.8	8.7	48.6	3.7
A77V	326,511	69.9	2.9	9.5	45.4	5.3
RIC	112,183	45.1	3.2	7.9	51.2	2.8

a Results from one set of duplicate infections; see Table S1 for accompanying statistical analyses.

b NLX.Luc.R- constructs; RIC, random integration control.

### CPSF6 interaction with retroviral capsids.

**FIG 5:**
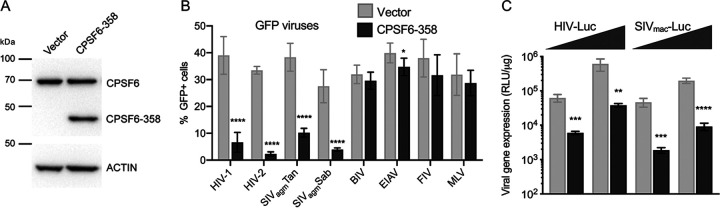
CPSF6-358 restriction assays. (A) Lysates of HEK293T cells transduced with empty or CPSF6-358 expression vector were probed with anti-CPSF6 (upper panel) or anti-β-actin (lower panel) antibody. The upper band in the CPSF6 immunoblot is endogenous CPSF6[551]. Numbers to the left of the blots mark migration positions of mass standards. (B) Infectivities of indicated GFP reporter viruses on vector versus CPSF6-358-expressing cells. (C) Infectivities of HIV-1 and SIV_mac_ Luc reporter constructs. Results in panel A are representative of those observed in two independent experiments; in panels B and C, results (average ± SD) compile data from at least four independent experiments, with each experiment conducted in duplicate. *, *P* < 0.05; **, *P* < 0.01; ***, *P* < 0.001; ****, *P* < 0.0001.

The interaction of CPSF6 with retroviral capsids was virologically assessed via CPSF6-358 restriction (33). Vector control HEK293T cells were infected with GFP viruses at approximate MOIs of 0.4 to 0.5 alongside cells expressing CPSF6-358 (Fig. 5A). As expected (33), MLV-GFP similarly infected control and CPSF6-358-expressing cells, and CPSF6-358 potently restricted infection by primate lentiviruses (Fig. 5B). Also as reported previously (33), CPSF6-358 did not significantly restrict FIV infection. While infection by BIV was similarly unaffected, CPSF6-358 mildly restricted EIAV (Fig. 5B). Consistent with prior findings (33), restriction of HIV-1 and SIV_mac_-Luc viruses was observed across a range of virus inocula (Fig. 5C).

**FIG 6:**
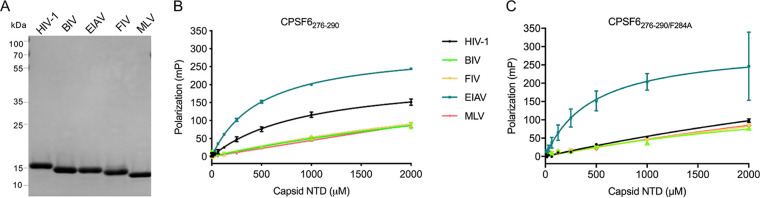
FP spectroscopy analysis of CPSF6-CA NTD interactions. (A) Electrophoretic analysis of indicated purified proteins. The gel was stained with Coomassie blue; numbers to the left demarcate mass standard positions. (B) FP measurements of CPSF6_276–290_ binding to the indicated capsid NTD protein. (C) FP measurements of CPSF6_276–290/F284A_ binding to indicated proteins. Panel B and C results are average ± SD for *n* = 2 independent experiments, with each experiment conducted in triplicate.

While results of CPSF6-358 restriction assays (33, 35) (Fig. 5) indicate lack of productive binding between human CPSF6 and FIV capsid, CPSF6_276–290_ peptide was previously shown to bind HIV-1, HIV-2, SIV_mac_, and FIV capsid NTD proteins with similar affinities (37). To reinvestigate this finding and expand the study to other nonprimate lentiviruses, the NTDs of BIV, EIAV, and FIV capsid protein were purified following their expression in Escherichia coli (Fig. 6A). Control proteins included the NTDs of HIV-1 and MLV capsid. Binding to fluorescently labeled CPSF6_276–290_ peptide was assessed by fluorescence polarization (FP) spectroscopy (44, 45).

We determined the binding constant 943 ± 86.5 μM for the HIV-1 capsid NTD-peptide interaction (Fig. 6B and Table 5), which agreed with the 872 μM *K_D_* (equilibrium constant) previously reported by FP (45). While the binding constants for BIV, FIV, and MLV proteins all exceeded 2 mM, which was the highest concentration of protein tested, the peptide bound the EIAV NTD with an apparent affinity of 496 ± 16.3 μM (Fig. 6B and Table 5). To assess the specificities of peptide interactions with HIV-1 and EIAV NTD proteins, a second version was synthesized with Phe substituted for Ala, corresponding to the F284A change in CPSF6[551] (Fig. S1B). The analogous F321A change in CPSF6-358 negated HIV-1 restriction (36), and ectopic CPSF6[551]F284A expression failed to complement the HIV-1 integration targeting defect of CKO cells (5, 8, 17). Because the CPSF6_276–290/F284A_-HIV-1 capsid NTD *K_D_* was >6 mM, the ∼940 μM dissociation constant for CPSF6_276–290_ is consistent with the known capsid-CPSF6 interaction (36, 37). In contrast, CPSF6_276–290/F284A_ displayed an apparent *K_D_* of 466 ± 135 μM for the EIAV capsid NTD (Fig. 6C and Table 5).

**TABLE 5:** *K_D_* values of CPSF6 peptide-capsid NTD interactions

Peptide	Capsid NTD protein	*K_D_* (μM)	SD^*a*^
CPSF6_276–290_	HIV-1	943	86.5
	BIV	>5,000	>3,000
	FIV	>4,000	>1,000
	EIAV	496	16.3
	MLV	>5,000	>2,000

CPSF6_276–290/F284A_	HIV-1	>6,000	>2,000
	BIV	>3,000	>2,000
	FIV	>7,000	>4,000
	EIAV	466	135
	MLV	>8,000	>4,000

a SD, standard deviation for minimally *n* = 2 independent experiments, with each conducted with triplicate measures.

## DISCUSSION

Our results clarify that the dramatic preference for HIV-1 to target SPADs for integration (5) applies to HIV-2 as well. In contrast, nonprimate lentiviruses targeted SPADs at comparatively low frequencies. FIV in particular disfavored SPADs in Jurkat T cells, and MLV, moreover, targeted SPADs at greater frequencies than the nonprimate lentiviruses studied here in both HEK293T and Jurkat T cells. Superenhancers are known to associate with nuclear speckles (46, 47), which we suspect accounts for MLV’s SPAD preference. In contrast to MLV, superenhancers are not preferred targets of bulk HIV-1 integration (5, 48). We conclude that the capsid-CPSF6 interaction underlies the dramatic difference in SPAD-tropic integration targeting between primate and nonprimate lentiviruses.

Our work clarifies that LEDGF/p75 plays similarly important roles in genic integration targeting of primate and nonprimate lentiviruses. As assessed by CPSF6-358 restriction as well as peptide binding to NTD proteins, CPSF6 did not meaningfully interact with FIV, BIV, or EIAV capsids. These data are consistent with prior observations that the artificial TRIM-CPSF6-358 construct failed to restrict nonprimate lentiviral infection (35). Amino acid sequence alignment of capsid proteins highlighted conservation of key residues for the interaction with CPSF6 across primate lentiviral proteins, with only a smattering of conservation at these positions among nonprimate lentiviruses (see Fig. S5 in the supplemental material). Because CPSF6 is highly conserved among mammals (Fig. S1B), we would speculate that substitutions of key contact residues among the capsid proteins underlie the affinity differences observed for primate versus nonprimate lentiviral proteins for interacting with CPSF6.

10.1128/mBio.02254-20.5 FIG S5 Sequence conservation among lentiviral capsid proteins. The alignment generated using Clustal Omega (version 1.2.3) (61) under default positions was visualized with ESPript 3.0 (65) (positions of amino acid homology boxed with homologous residues in red type; identical residues highlighted by red background). The NTD and C-terminal domain (CTD) of HIV-1 capsid are marked; dotted line, connecting linker. HIV-1 capsid residues that when mutated resulted in >5-fold reductions in binding to CPSF6 peptide (37, 45) or ≥70% reduction of binding to endogenous CPSF6 protein by capsid nanotube copelleting (33, 43) are noted by inverted triangle. MLV capsid was omitted from the alignment due to the inability for Clustal Omega to meaningfully incorporate it. Inspection of a published sequence alignment (U. K. von Schwedler, T. L. Stemmler, V. Y. Klishko, S. Li, et al., EMBO J 17:1555–1568, 1998) revealed the following MLV capsid residues (in single-letter code) at analogous HIV-1 capsid positions: Asn57, N; Met66, G; Asn74, I; Ala77, Q, Thr108, T; Lys182, E. Download FIG S5, PDF file, 0.03 MB. Copyright © 2020 Li et al. 2020 Li et al. This content is distributed under the terms of the Creative Commons Attribution 4.0 International license.

Because CPSF6-358 failed to appreciably restrict EIAV infection (35) (Fig. 5), it is unclear why CPSF6 depletion reduced EIAV genic integration by ∼5% in both HEK293T and Jurkat T cells. Although these were mild differences compared to the ∼22% reductions in genic targeting conferred via LKO, CPSF6 depletion failed to significantly impact genic integration targeting by MLV, lending specificity to the EIAV responses. Because CPSF6_276–290_ and CPSF6_276–290/F284A_ peptides bound EIAV capsid NTD protein at similar affinities, we cannot exclude an interaction between EIAV capsid and CPSF6, although this would have to fundamentally differ from the known interaction with HIV-1 capsid (36, 37, 45, 49). At the same time, caution should be exercised in interpreting capsid NTD binding data. For example, we could not recapitulate the ∼0.24 mM *K_D_* previously attributed by isothermal calorimetry for CPSF6_276–290_-FIV capsid NTD binding (37), indicating that some NTD protein preparations may be uncharacteristically sticky. Because CPSF6 peptide binds HIV-1 capsid hexamers approximately 10-fold more efficiently than the NTD (45), assays with higher-order EIAV capsid assemblies and/or full-length CPSF6 protein could potentially be explored. Our results at the same time do not address indirect effects of CPSF6 depletion, for example, via dysregulating the functionality of novel genic targeting cofactors, on EIAV integration. The observation that SPAD-proximal integration by EIAV was unaffected by CPSF6 depletion highlights the role of CPSF6 in primate lentiviral integration targeting.

**FIG 7:**
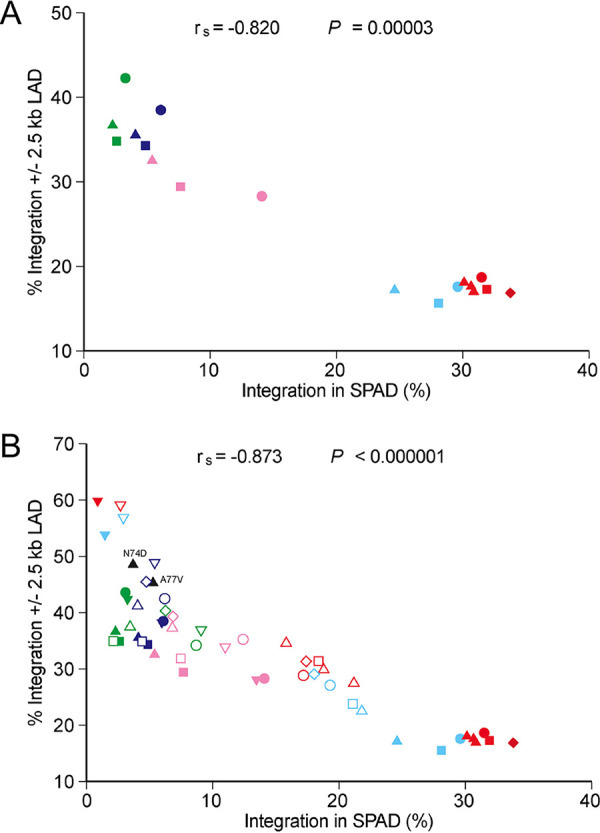
Spearman rank analyses of lentiviral integration targeting. (A) Correlation of lentiviral LAD- versus SPAD-tropic integration values in WT HEK293T cells (filled circles) and Jurkat T cells (WT, upright filled triangles; NTC, filled squares; NT, filled diamonds) (Tables 1 to 4 and Fig. S3E). Resulting *r_s_* and *P* values are indicated. Data point color representation: red, HIV-1; light blue, HIV-2; blue, BIV; pink, EIAV; green, FIV. (B) Same as in panel A, expanded to include CPSF6 and/or LEDGF/p75 depletion conditions (open circles, LKO HEK293T cells; inverted filled triangles, CKO HEK293T cells; inverted empty triangles, DKO HEK293T cells; empty upright triangles, LKO Jurkat T cells; empty diamonds, IBD^−/−^ Jurkat T cells; empty squares, CPSF6 knockdown [CKD] Jurkat T cells). Data from HIV-1 N74D and A77V capsid mutant viruses are labeled (black triangles). MLV data were omitted from these analyses.

Regions of transcriptional activity segregate to nuclear interior and peripheral hot zones (50). Although the results of some image-based studies have indicated that HIV-1 under basal infection conditions prefers to integrate into chromatin in association with the periphery (7, 51, 52), results of other studies indicated that HIV-1 integration occurs more evenly dispersed throughout the nucleus (5, 8, 22, 53). In our hands, peripheral nuclear targeting by image-based measures positively tracked with LAD-associated integration (8). Viruses unable to interact with CPSF6 due to amino acid substitutions in capsid or via factor depletion accumulated at the nuclear periphery (18–22, 54) and gained significant preferences for LAD-proximal integration (8) at the expense of SPAD targeting (5) (Fig. 3 and 4). Plotting SPAD- versus LAD-proximal integration frequencies revealed a significant inverse correlation (Fig. 7A) (*P* = 3 × 10^−5^) for lentiviruses that was further enhanced by incorporating conditions of CPSF6 and/or LEDGF/p75 depletion (Fig. 7B) (*P* < 10^−6^). Although additional image-based work would be needed to make firm conclusions, our results indicate that nonprimate lentiviruses may very well preferentially target the peripheral region of the nucleus for integration under basal infection conditions.

In addition to integration targeting, CPSF6 influences HIV-1 PIC nuclear import (19, 21, 22, 54–56). Although precise nuclear import mechanisms await clarification, nonprimate lentiviruses seemingly rely on host cofactors that are distinct from those used by HIV-1 (33, 57). Because CPSF6 binding-defective HIV-1 capsid proteins were largely excluded from the nucleus (22, 58), nonprimate lentiviruses could plausibly shed their capsids prior to nuclear entry. Differential primate versus nonprimate lentiviral PIC nuclear import requirements are consistent with a unique role for CPSF6 in primate lentiviral integration targeting, reinforcing previously suggested mechanistic links between nuclear import and integration (59, 60). Indeed, we would conjecture that CPSF6 uniquely links primate lentiviral PIC nuclear import and SPAD integration targeting. We would, moreover, conclude that the lentiviral replication fitness advantage provided by the capsid-CPSF6 interaction (42, 43) evolved in primate species.

## MATERIALS AND METHODS

### Protein sequence alignments.

The following sequences were extracted from the National Center for Biotechnology Information database (accession code in parentheses): H. sapiens PC4 and SRSF1-interacting protein (PSIP1) isoform 2 (NP_001121689.1) and CPSF6 isoform 1 (NP_008938.2), M. mulatta PSIP1 isoform X1 (XP_014973138.1) and CPSF6 isoform X4 (XP_015007722.1), *E. caballus* PSIP1 isoform X1 (XP_023482789.1) and CPSF6 isoform X4 (XP_023499753.1), *F. catus* PSIP1 isoform X1 (XP_023097965.1) and CPSF6 isoform X4 (XP_023112991.1), *M. musculus* PSIP1 isoform 1 (NP_598709.1) and CPSF6 isoform 2 (NP_001013409.1), *B. taurus* PSIP1 (NP_001193405.1) and CPSF6 (NP_001071574.1), HIV-1_NL4-3_ (U26942.1), HIV-2_ROD_ (X05291.1), SIV_agm_Tan-1 (U58991.1), EIAV (M16575.1), FIV (M25381.1), BIV (NC_001413.1), and MLV (J02255.1).

To visualize percent identity, sequences were aligned in Clustal Omega (version 1.2.3) (61) using default parameters. Alignments for Fig. S1 in the supplemental material were imported into Jalview (v. 2.10.5) (62), and point-by-point percent conservation files were imported into R (v. 3.5.1). A rolling average was calculated over a window length of 5 residues with the package zoo (v. 1.8.4) (63), and resulting plots were generated with ggplot2 (v. 3.1.0) (64) and cowplot (v. 0.9.4). Alignments of primary amino acid sequences were visualized using ESPript 3.0 (65).

### Plasmid DNAs.

Plasmid psPAX2 was obtained from the NIH AIDS Reagent Program while plasmids pIRES2-eGFP (13), pNLX.LucR-U3-tag (66), and pCG-VSV-G (67) were previously described. Other plasmids that encoded single-round HIV-1 (68), HIV-2 (69), SIV_agm_Sab, SIV_agm_Tan (70), SIV_mac_ (71), BIV (72), EIAV (73), FIV (74), and MLV (75) GFP constructs as well as HIV-1 (WT, N74D) (76, 77), SIV_mac_ (78), MLV (13), and FIV (79) Luc reporter viruses were also previously described. The A77V change in capsid was introduced into the pNLX.Luc.R-.ΔAvrII (77) *gag* gene using primers AE7236/AE7237 and NEBuilder HiFi DNA assembly master mix as recommended by the manufacturer. To make plasmid pEIAV-Luc, the luciferase gene from pHI-Luc (76) was amplified using primers AE7354 and AE7357. The resulting amplicon was digested with Acc65I and NotI, followed by ligation with Acc65I/NotI-digested pEIAV-GFP DNA. See Table S2 for additional reagent details including retroviral transfer vector promoters and oligonucleotide sequences.

10.1128/mBio.02254-20.7 TABLE S2 Retroviral vector and oligonucleotide reagents. Download Table S2, XLSX file, 0.02 MB. Copyright © 2020 Li et al. 2020 Li et al. This content is distributed under the terms of the Creative Commons Attribution 4.0 International license.

Plasmids for expressing NTDs of HIV-1, EIAV, FIV, and MLV capsid proteins in E. coli were previously described (35). The corresponding BIV expression vector was made by amplifying the capsid coding region of pBH2 (72) with primers AE7572/AE7573, digesting the DNA with NdeI and BamHI, and then ligating to NdeI/BamHI-digested pET-22b. The resulting construct encoded a hexahistidine (His_6_) tag appended onto the C terminus of the BIV capsid NTD protein. The sequences of all plasmid DNAs built using PCR here were verified by Sanger sequencing.

### Cell lines and growth conditions.

WT HEK293T cells as well as isogenic LKO, CKO, and DKO derivatives were described previously (17, 41). Single cell CKO and DKO clones B8 and F6, respectively, were used in this work. Vector control and CPSF6-358-expressing HEK293T cells were previously described (33). HEK293T cells were cultured at 37°C in Dulbecco’s modified Eagle’s medium containing 10% (vol/vol) fetal bovine serum (FBS), 100 IU/ml penicillin, and 100 μg/ml streptomycin (DMEM) in humidified incubators in the presence of 5% CO_2_.

WT Jurkat T cells as well as previously described LKO and IBD^−/−^ derivatives, referred to respectively as PSIP1^−/−^ and IBD^−/−^ clone 1 in reference 41, were cultured in RPMI 1640 containing 10% FBS, 100 IU/ml penicillin, and 100 μg/ml streptomycin (RPMI). Additional LKO Jurkat T cell lines were generated as follows. Single guide RNA (sgRNA) 5′-AAGUGAAGCAAGUUCAUCCA-3′ targeting exon 12 of the *PSIP1* gene was purchased from GenScript as a custom pLentiCRISPRv2 vector (pLEDGF_7125). To assemble lentiviral transduction particles, HEK293T cells were cotransfected with pLEDGF_7125, psPAX2, and pCG-VSV-G at respective mass ratios of 0.53:0.33:0.14 using Effectene *in vitro* transfection reagent (Qiagen). CRISPR-Lenti NT control plasmid CRISPR12 (Sigma-Aldrich) replaced pLEDGF_7125 in separate transfections. Virus-containing supernatant harvested at 48 h posttransfection was filtered through 0.45-μm filters and stored at −80°C. Jurkat T cells were transduced with thawed supernatant in 24-well plates by spin-inoculation at 200 × *g* for 1 h at room temperature. After 24 h, puromycin (Thermo Fisher Scientific) was added to 0.25 μg/ml final concentration. At 4 days postransduction the puromycin concentration was increased to 0.3125 μg/ml. After a week of puromycin selection, cells were frozen and stored at −135°C. Thawed cells were cultured in selection medium (RPMI 1640, 20% FBS, 20% Jurkat T cell conditioned medium, 100 IU/ml penicillin, 100 μg/ml streptomycin, 0.3 to 0.5 μg/ml puromycin) for 3 weeks prior to single-cell cloning by limiting dilution in 96-well plates. Cell clones propagated in selection medium were phenotypically analyzed for LEDGF/p75 content by immunoblotting and genotyped by Sanger sequencing of PCR amplicons.

### Virus production and infectivity assays.

Single-cycle GFP and Luc reporter viruses were produced by cotransfection of HEK293T cells in 10-cm dishes with 15 μg total of various ratios of virus production plasmids using PolyJet DNA transfection reagent (SignaGen Laboratories). Culture supernatants after 48 h were clarified by passage through 0.45-μm-pore syringe filters and ultracentrifuged for 2 h at 26,000 rpm in an SW32-Ti rotor. Virus pellets were resuspended in DMEM, frozen in aliquots, and stored at −80°C. Aliquots were thawed only once for infection assays. Concentrations of retroviral particles in mU/ml were determined using a TaqMan-based product-enhanced reverse transcriptase (Taq-PERT) real-time PCR assay (80).

Infections were conducted in duplicate in 24-well plates (10^5^ cells/well). After 6 to 8 h, medium was replaced with fresh DMEM or RPMI, and cells were harvested at 48 h from the start of the experiment. GFP reporter viral MOIs were determined as percentage of GFP-positive cells using a FACSCanto flow cytometer (BD Biosciences). Luc reporter viruses unless otherwise noted were inoculated at 0.5 μU RT per cell, and cells were lysed in passive lysis buffer (Promega) by freezing overnight at −80°C and thawing at 37°C for 30 min. Cell lysates were centrifuged at 17,500 × *g* for 8 min, and supernatants were analyzed in triplicate for luciferase activity. Relative light units (RLU), determined by luminometer (Berthold Technologies), were normalized to total protein concentration as measured by Pierce BCA protein assay kit (Thermo Fisher Scientific).

### Genotyping of Jurkat LKO cells.

Genomic DNA isolated using the Zymo Research Quick gDNA Microprep kit according to the manufacturer’s instructions was amplified by primers AE7920 and AE7921 using Phusion polymerase to capture the sgRNA-targeted site within *PSIP1*. Amplified products were assembled with XhoI/EcoRI-digested pIRES2-GFP using NEBuilder HiFi DNA assembly master mix. Recombinant plasmids recovered from at least 10 bacterial colonies per cell clone were sequenced by Sanger sequencing. Predicted amino acid sequences of mutated LEDGF/p75 protein within individual cell clones were deduced from resulting DNA sequences. Such analyses revealed at most two mutant *PSIP1* alleles per cell clone.

### CPSF6 depletion in Jurkat T cells.

CRISPR RNAs (crRNAs) targeting *CPSF6* (UUCAGAUCCAACACCAACAA), NTC (GAUACGUCGGUACCGGACCG), and *trans*-activating crRNA (tracrRNA; proprietary sequence), as well as Cas9-NLS protein, were purchased from Dharmacon. Details of crRNP complex formation were as described previously (81). Briefly, to form 20 μM crRNP complex, 2.5 μl each of 160 μM tracrRNA and 160 μM crRNA were mixed and incubated at 37°C for 30 min, followed by addition of 5 μl of 40 μM Cas9-NLS and incubation at 37°C for 15 min.

Jurkat T cells (1 × 10^6^) resuspended with nucleofection buffer containing the required supplement from the Cell Line Nucleofector kit V (Lonza) were mixed with 20 μM crRNP and electroporated by nucleofector I according to the manufacturer’s instructions. After electroporation, cells were plated in 6-well plates containing 2 ml prewarmed RPMI and incubated for minimally 3 days to allow recovery prior to immunoblotting and virus infection.

### Western blot analyses.

Cells were lysed for immunoblotting as previously described (17) except that the NaCl concentration was 400 mM in the lysis buffer. Total protein was measured by the BCA assay kit (Pierce), and equal amounts (20 μg) were separated through 12% polyacrylamide gels under denaturing conditions. Gels were transferred to polyvinylidene fluoride membranes at 20 V for 30 min using a Trans-Blot SD semidry electrophoretic transfer cell (Bio-Rad). The following antibodies were used for immunoblotting: anti-CPSF6 (ab175237; Abcam), anti-LEDGF/p75 (A300-848A; Bethyl Laboratories), antiactin-horseradish peroxidase (HRP) conjugated (A3854-200UL; Sigma-Aldrich), and horseradish peroxidase-conjugated anti-rabbit IgG secondary antibody (Dako).

### Protein expression and purification.

HIV-1, EIAV, FIV, and MLV capsid NTD proteins with C-terminal His_6_ tags were expressed in E. coli BL21(DE3) and purified as described previously (35). The corresponding BIV protein was similarly purified by capture on Ni-nitrilotriacetic acid (NTA) resin (Thermo Scientific) followed by size exclusion chromatography. Briefly, shaker flasks of BL21(DE3) cells were grown in LB medium at 37°C to *A*_600_ of 0.6 prior to induction with 0.5 mM isopropyl-β-d-1-thiogalactopyranoside for 3 to 4 h, after which cells were harvested and resuspended by sonication in lysis buffer (50 mM Tris, pH 8.0, 100 mM NaCl, 30 mM imidazole). Following centrifugation at 40,000 × *g* for 1 h at 4°C, the supernatant was incubated with 2 ml Ni-NTA agarose beads (Qiagen) overnight at 4°C. After extension washing with lysis buffer, protein was eluted with buffer containing 50 mM Tris, pH 8.0, 100 mM NaCl, and 500 mM imidazole. NTD-containing fractions were further purified by gel filtration on a Superdex-200 column (GE Healthcare) in 50 mM Tris (pH 8.0) and 50 mM NaCl. NTD protein concentrated by ultrafiltration using Amicon ultracentrifugal filters with a 3,000-molecular-weight cutoff (Millipore) was flash-frozen in liquid N_2_ and stored at −80°C.

### FP spectroscopy.

N-terminal fluorescein isothiocyanate (FITC)-Ahx fluorescently tagged CPSF6_276–290_ (PVLFPGQPFGQPPLG) and CPSF6_276–290F284A_ (PVLFPGQPAGQPPLG) peptides were synthesized at GenScript. Increasing concentration (from 2 μM to 2 mM) of capsid NTD proteins were mixed with 150 nM labeled peptide in 10 μl assay buffer (50 mM Tris-HCl, pH 8.0, 50 mM NaCl) in black 384-well nonbinding surface coated microplates (Corning). Levels of FP in millipolarization (mP) were measured on a SpectraMax M5 microplate reader (Molecular Devices) using respective excitation and emission wavelengths of 488 nm and 526 nm. Data were analyzed using GraphPad Prism 7 and fitted to a one-site specific binding model.

### Integration site sequencing.

VSV-G pseudotyped reporter viruses were treated with Turbo DNase (Life Technologies) at 37°C for 1 h prior to infection, and genomic DNA at 5 days postinfection was extracted from infected cells using the DNeasy blood and tissue kit (Qiagen). Integration site data were derived from two different sets of virus infections, either independent experiments conducted on separate days (Table 1) or duplicate infection samples conducted in parallel (Tables 2 to 4). Resulting integration sites were demultiplexed to yield unique integration site usage across experimental replicates. The Fig. S3E data were derived from one set of virus infections.

Integration libraries were prepared by LM-PCR essentially as described previously (82, 83) with modifications to accommodate previously unstudied viruses. Genomic DNA (2 to 10 μg) from MLV- and HIV-1-infected cells in Tables 1 to 4 was digested with MseI and BglII overnight at 37°C; for the experiment in Fig. S3E, the DNA was digested with AvrII, NheI, SpeI, and BamHI. DNA from HIV-2-, FIV-, BIV-, and EIAV-infected cells was digested with MseI with additional respective enzymes KpnI, SacI, HindIII, and BbvcI. Digested DNA was purified and ligated overnight at 12°C with asymmetric double-stranded linkers containing 5′-TA (Tables 1 to 4) or 5′-GATC (Fig. S3E) overhangs. After purification, ligated DNA was subjected to PCR using viral U5 (Tables 1 to 4) or EIAV-U3 (Fig. S3E) and linker-specific primers. Following purification, heminested second-round PCR was performed using nested virus-specific primers and the same linker-specific primer. Linker-specific primers and second-round virus-specific primers contained sequences at their 5′ ends for Illumina-compatible DNA sequencing (see Table S2). LM-PCR products were subjected to 150-bp paired-end sequencing on Illumina platforms either at the Dana-Farber Cancer Institute Molecular Biology Core Facilities or at Genewiz.

### Bioinformatics.

Illumina reads were processed and integration sites were mapped essentially as previously described (17, 66, 82). Briefly, read 1 of the paired-end sequences contained viral information linked to host DNA whereas the second read contained the linker and opposing end of the captured host fragment. Reads that contained terminal 15-bp matches to viral U5 (5′-GGAAAATCTCTAGCA, 5′-GGAAAATCCCTAGCA, 5′-GTTCGAGATCCTACA, 5′-GAAGAACACCCAACA, 5′-GCCGAGAACTTCGCA, and 5′-AGCGGGGGTCTTTCA for HIV-1, HIV-2, EIAV, BIV, FIV, and MLV, respectively) or HIV-1 U3 (5′-GAATTAGCCCTTCCA) and the linker were selected. After trimming viral sequences from read 1 and linker sequences from read 2, both reads were aligned to human genome build hg19 using BWA-MEM with paired-end option (84). Alignments were filtered by SAMtools to remove unmapped, secondary alignments and low-mapping-quality scores (85). Reads filtered for <900-bp separation between integration and linker ligation sites were selected and converted into BED format. The left interval of the BED format was determined by adding 2 bp to the site if the site was on the positive strand and by subtracting 2 if the site was on the negative strand. Similarly, the right interval of the BED format was obtained by adding 3 to the site if the site was on the positive strand and by subtracting 3 if the site was on the negative strand. The resulting information was used to assess integration site distributions with respect to various genomic features using BEDtools (86) as described previously (5, 8, 82).

Chromosomal regions that lie within 500 nm of nuclear speckles are defined as SPADs (50). The SPAD data set was reconstructed using Bowtie2 (87) as tyramide signal amplification-sequencing scores greater than the 95th percentile (50). From 1,547,458 resulting SPADs (each 100 bp in length), 63.5% mapped to genes. The average intron content of SPAD-associated genes, 9.8, which was determined as described previously (16), was similar to the average 9.6 intron-per-gene content of all human genes.

To plot integration normalized for gene length, targeted genes were divided into 10 equal bins and the number of integration sites per bin was normalized by the total number of genic integration sites for that experiment.

### Statistical analyses.

Statistical significance was assessed using one-way or two-way ANOVA (critical *P* < 0.05 was considered significant) in GraphPad Prism Version 7 (GraphPad Software) for the following figure panels: Fig. 1B and C, Fig. 2B to E, Fig. 4D, Fig. 5B and C, Fig. S2, and Fig. S3D. Fisher’s exact test was used for comparisons of integration site distributions in Python with *P* value calculated by the Stats module of the Scipy package (scipy.stats). Average number of genes per Mb was analyzed in R using Wilcoxon sum rank test. Two-tailed Spearman correlation (*r_s_*) analyses and *P* value determinations were performed using GraphPad Prism 8.

### Data availability.

Integration site sequences have been deposited in the National Center for Biotechnology Sequence Read Archive under accession code PRJNA647337.
