# Supplementary figures and images for: CPSF6-Dependent Targeting of Speckle-Associated Domains Distinguishes Primate from Nonprimate Lentiviral Integration
Source: mBio. 2020 Sep 29;11(5):e02254-20. doi: 10.1128/mBio.02254-20 (PMC7527728; doi:10.1128/mBio.02254-20)

Figure S1

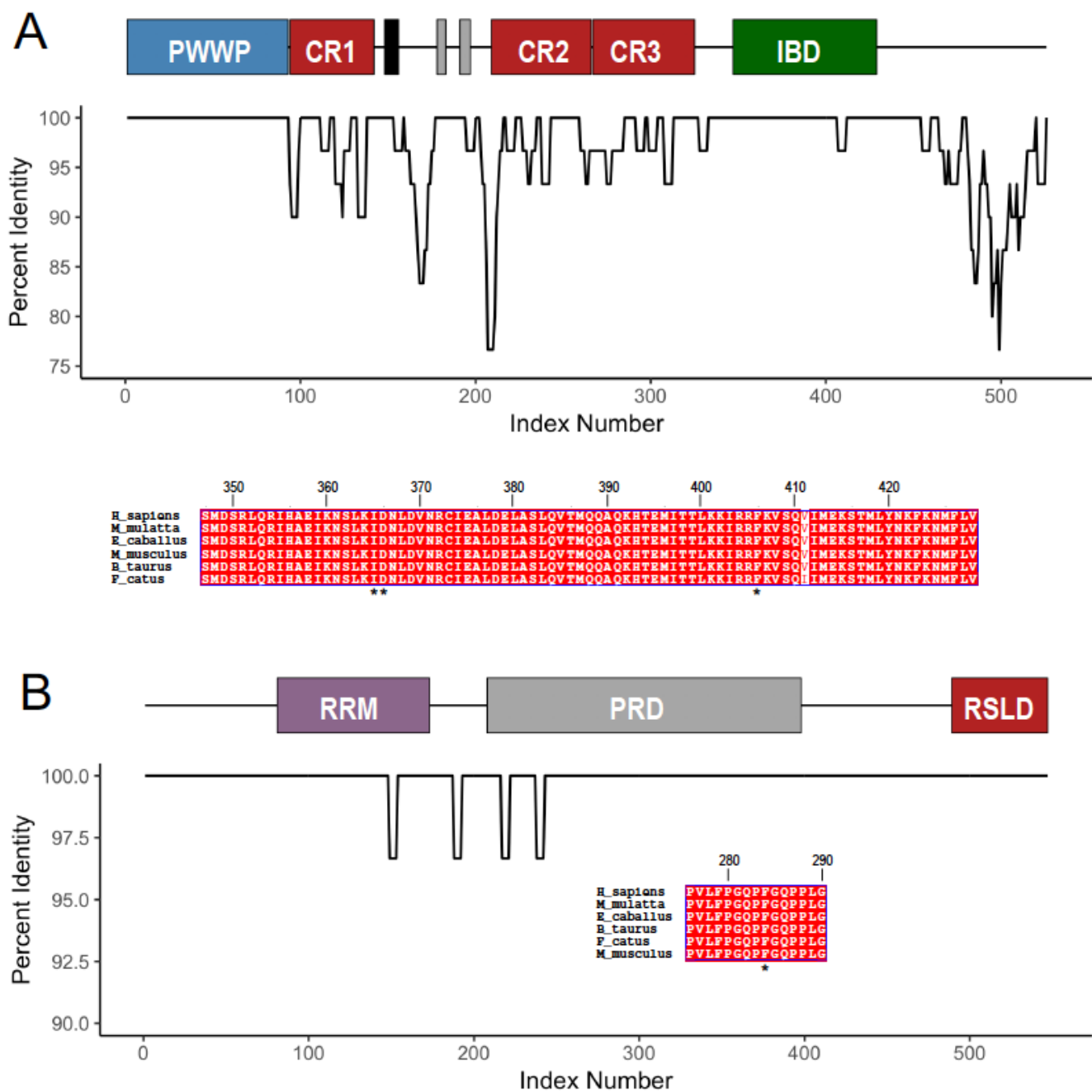

Supplement: FIG S1 [file mBio.02254-20-sf001.pdf]

Figure S2

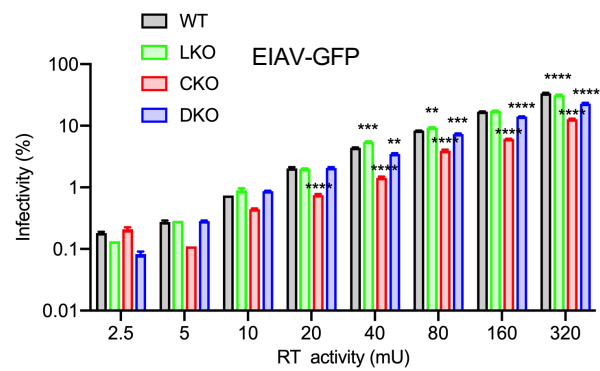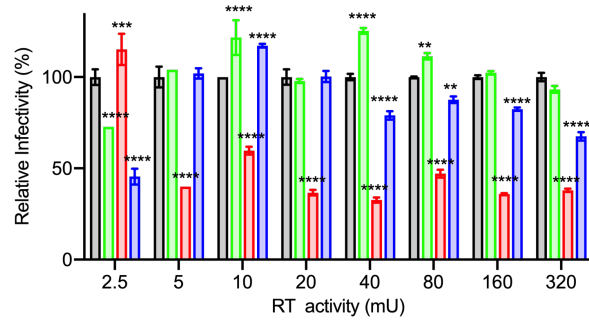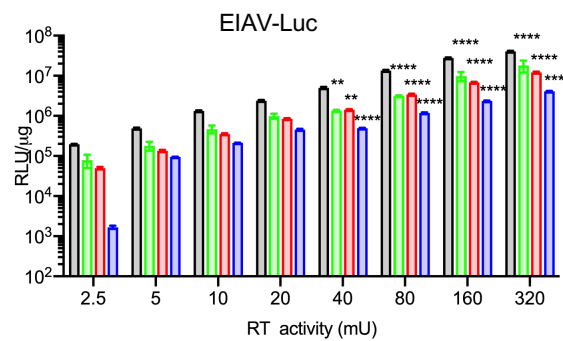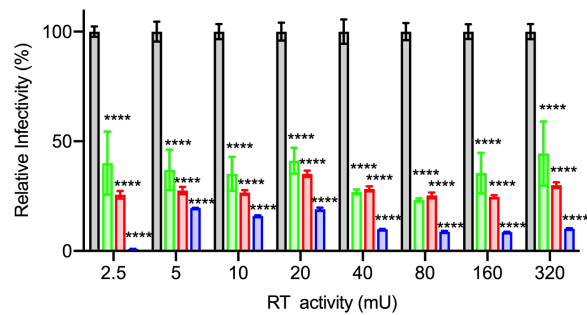

Supplement: FIG S2 [file mBio.02254-20-sf002.pdf]

Figure S3

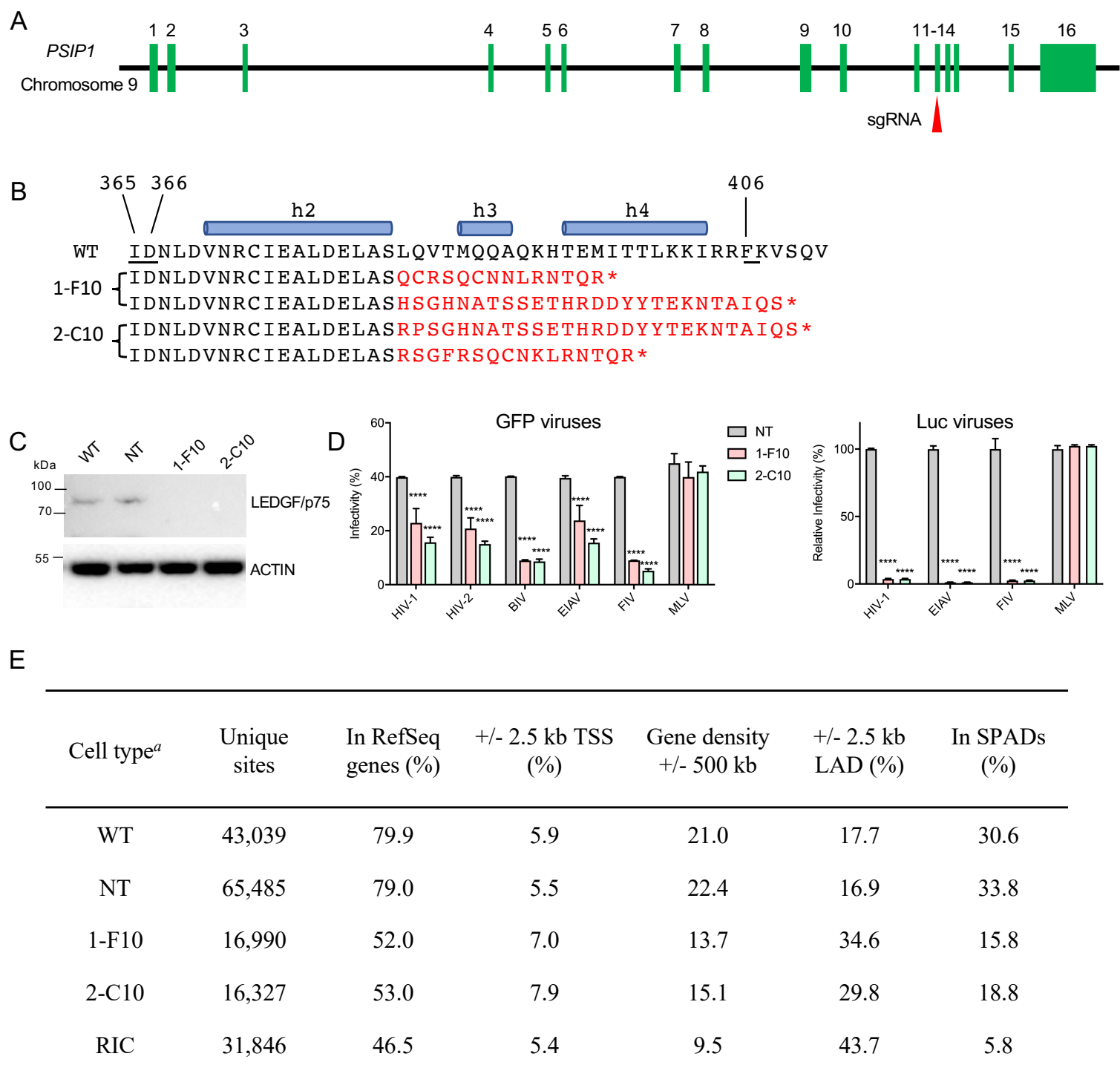

<sup>a</sup>WT, wild type Jurkat T cells; NT, non-targeting; RIC, random integration control

Supplement: FIG S3 [file mBio.02254-20-sf003.pdf]

Figure S4

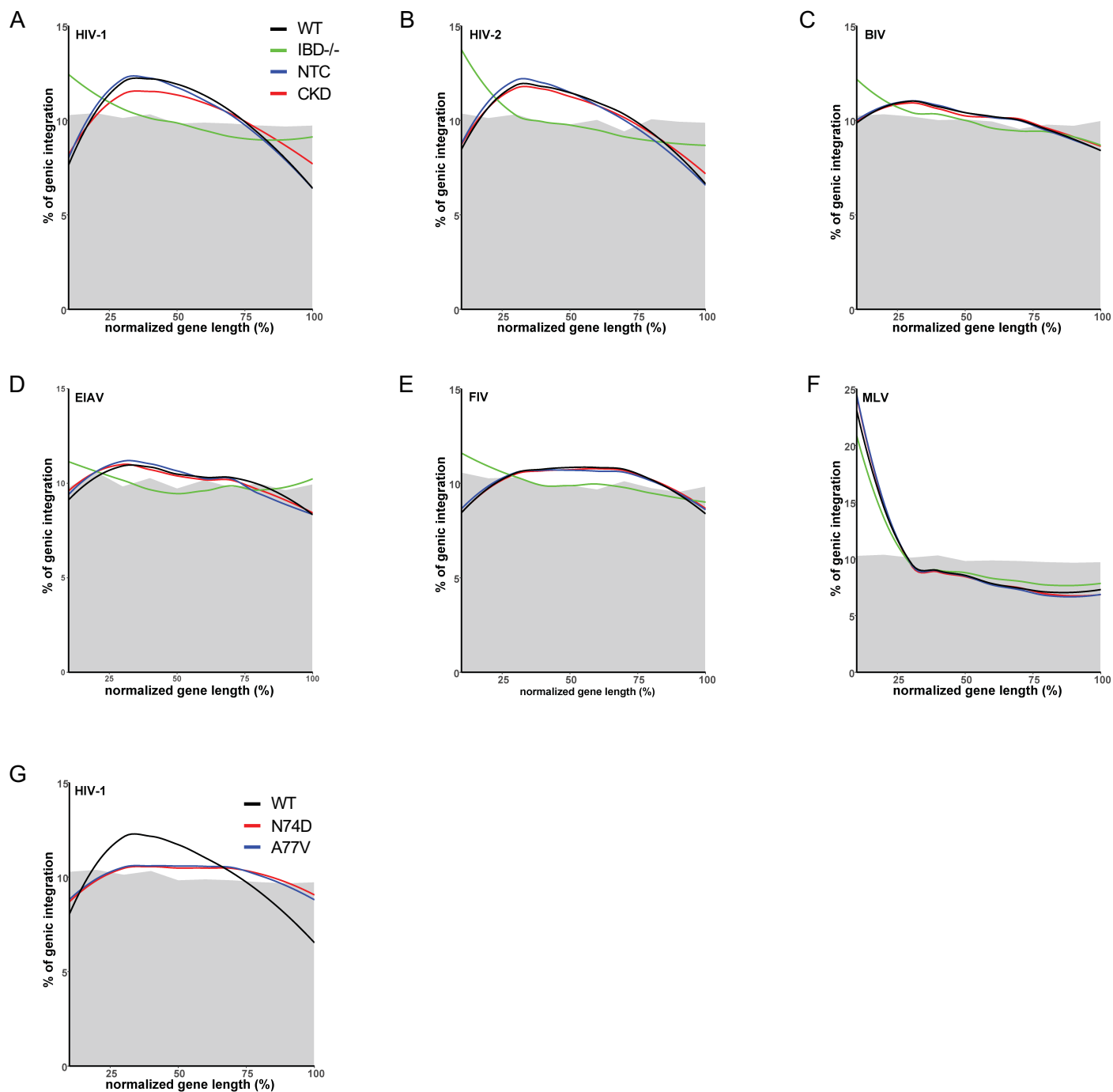

Supplement: FIG S4 [file mBio.02254-20-sf004.pdf]

Figure S5

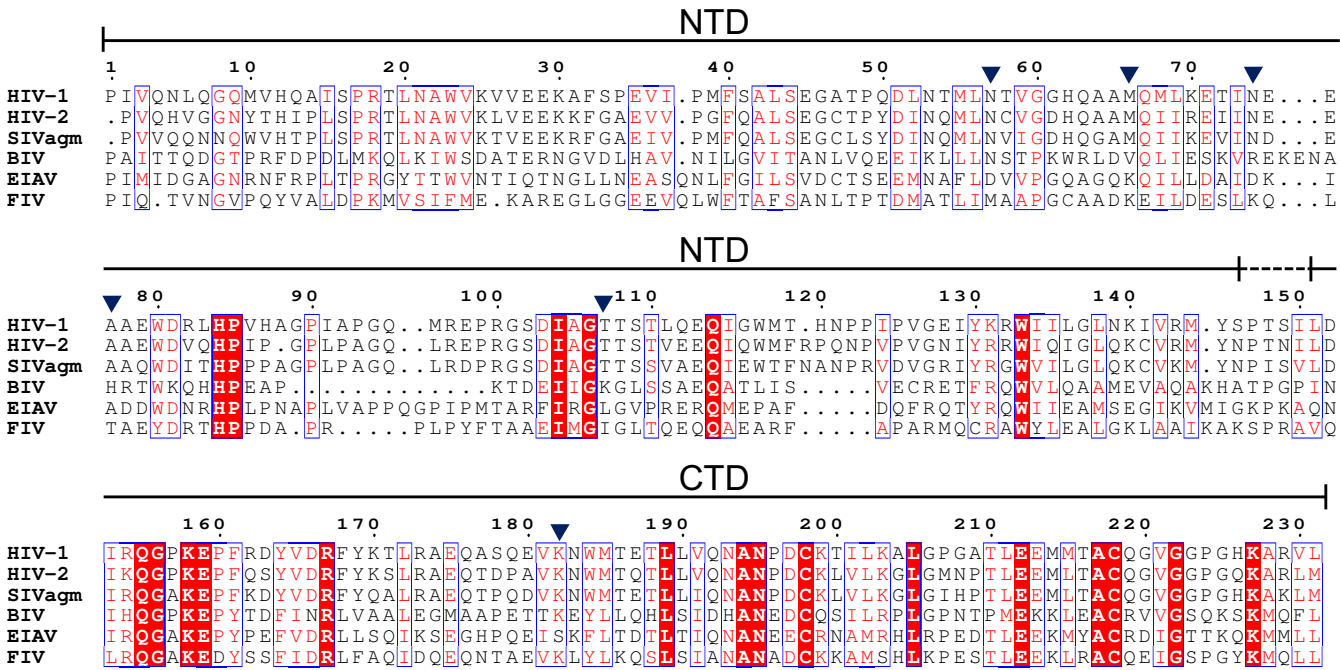

Supplement: FIG S5 [file mBio.02254-20-sf005.pdf]
